# Translation and validation of the Comfort Behaviors Checklist in hospitalized children with chronic diseases

**DOI:** 10.1186/s12887-023-04451-x

**Published:** 2023-12-09

**Authors:** Sara Valadkhani, Sima Hejazi, Azam Shirinabadi Farahani

**Affiliations:** 1 Department of Pediatric Nursing, School of Nursing and Midwifery, Shahid Beheshti University of Medical Sciences, Tehran, Iran; 2 Department of Nursing, Bojnurd Faculty of Nursing, North Khorasan University of Medical Sciences, Bojnurd, Iran; 3 Department of Pediatric Nursing, School of Nursing and Midwifery, Shahid Beheshti University of Medical Sciences, Tehran, Iran

**Keywords:** Comfort, Validation, Chronic disease, Children, Comfort Behaviors Checklist

## Abstract

**Background:**

Different tools have been developed to measure patients' comfort. This study aims to translate, validate, and apply the Comfort Behaviors Checklist to hospitalized children with chronic diseases.

**Methods:**

Validity and reliability are assessed using face and content validity, construct validity (known-groups technique and Principal Component Analysis), internal consistency, and inter-rater reliability. The study takes place in a children's hospital in Iran, involving 220 children aged 4 to 6.

**Results:**

The Comfort Behaviors Checklist demonstrates acceptable face and content validity. Construct validity is supported by the lack of correlation between behavioral comfort scores in known groups. The Principal Component analysis results in five components, explaining 70.39% of the total variation. The checklist exhibits acceptable reliability, with a total Cronbach's alpha coefficient of 0.86 and an intraclass correlation coefficient of 0.835.

**Conclusion:**

The Comfort Behavior Checklist is a valid and reliable tool for assessing the level of comfort in Iranian children with chronic diseases.

## Introduction

Children make up about 22.2% of the world's population [1], and 37.5% of them deal with chronic diseases [2, 3]. Chronic diseases have the highest mortality rate after accidents, with about 77% frequency in low- and middle-income countries [4]. Due to the physical problems and long-term treatment of chronic diseases, the child and family are exposed to psychological and mental injuries, and the individual's well-being is affected [5, 6].

Nurses need to provide comfort for the patient from a holistic point of view and special attention to psychological dimensions as well as spiritual and religious issues [7, 8]. Today, when speaking about patients' "comfort" in nursing practices, the attention of the healthcare system workers is drawn to patients' pain. However, providing comfort for children and their families is multidisciplinary and intercultural knowledge; when provided, nurses cherish the mutual effect [9].

The concept of "comfort" relates to a state of physical ease and relief from pain or limitation or relieving or reducing a person's feelings of sadness, well-being, and pleasant lifestyle [10]. Having comfort in any dimension of human life is a goal and is considered an achievement. Feeling comfort is emphasized by everyone in every field; in architecture by creating a sense of comfort in the design of houses [11], in technology with the advent of smartphones [12] even in sports and sportswear [13]. Thus, the meaning of comfort for people can be different, and it is described individually [14].

The theory of comfort is one of the theories of middle-range nursing that includes the concept of complete comfort on the one hand and suffering on the other. Kolcaba, who first proposed the theory of comfort in 1990, defined it as the basic human need, for three types of Comfort, which are liberation, ease and transcendence. In the first type, liberation is a situation that needs to be addressed and in which needs are met. The second type is calmness and the feeling of satisfaction and satisfaction when the process is easy for the person. In the final stage of transcendence and the third, the person overcomes problems and is released from enduring pain [14]. She believed that comfort accelerates the patient's recovery time [15].

One of the nurses' duties is to use appropriate nursing models to achieve the goals of health care [16], and model-based approaches mainly emphasize observation [17]. Therefore, the existence of a list of observable behaviors and identifying the presence or absence of comfort can help nurses achieve healthcare goals [18]. In 1994, Kolcaba developed and published a list of observable and identifiable behaviors based on comfort theory to determine the presence or absence of comfort which is called the Comfort Behavior Checklist [18]. The checklist, which has five dimensions and 30 behavioral modes, is scored by the Likert scale (score 1 (lowest score) to score 4 (highest score)). In this checklist, a score of zero or NA refers to a situation in which that behavior or item is not expected of the patient; For example, if the patient is asleep, using meaningful words is not expected to make sense. The dimensions of the Comfort Behaviors Checklist include vocalization, motor signs, performance, facial expression, and other symptoms. An essential point in the Comfort Behaviors Checklist is to pay attention to other patient conditions. Therefore, the patient's behavioral comfort level is measured and compared before and after the nursing procedure or in a time range [9].

Different tools have been developed to measure patients' comfort; The Comfort Scale was designed by Ambuel in 1992 and used for children in intensive care units [19]. Another instrument used for children aged 0 to 3 years was the Behavioral Comfort Scale, developed by Van Dijk in 2000 [20]. In 2014, Safavi Bayat et al. examined the correlation between the health status of patients with Acquired immunodeficiency syndrome (AIDS) and their comfort [21] and used Kolcaba's General Comfort Questionnaire. Payami Bosari also used the questionnaire to evaluate the effect of back massage on the comfort of patients with irritable bowel syndrome [22]. Kolcaba has also developed other tools to measure people's comfort level, including the General Comfort Questionnaire, Advanced Directives Comfort Questionnaire, Childbirth Comfort, Numerical Rating of Comfort, and Peri Anesthesia [18].

The "Comfort Behaviors Checklist" can be used for children only by observing. No study has been conducted to measure the comfort of hospitalized children using valid and reliable tools in Iran. Knowing that providing comfort accelerates the recovery time in the patient [15], the present study was conducted to translate, validate, and apply the "Comfort Behaviors Checklist" in hospitalized children with chronic diseases. Nurses and health researchers in Iran can use this tool to assess the level of comfort in children by only observing their behavior and without the need for verbal communication, which is one of the challenges of hospitalization of children, to promote the nurse-patient relationship.

## Method

Sampling for the current methodological study was performed in the time range between August to March 2021 using a demographic questionnaire and the "Comfort Behaviors Checklist”. The translation and psychometrics of the checklist were based on the method proposed by Wild et al. in 2005 [23, 24]. After translating and back-translating the checklist, content and face validity, construct validity, and stability reliability were determined to analyze the psychometric characteristics of the instrument [23, 25]. The average time required to fill out the checklist was 4 min.

### Translation

Permission was first obtained from the developer of the Comfort Behaviors Checklist, Ms. Kolcaba, through email. The checklist was then translated into Persian by two people who were fluent in English separately so that there was no change in the meaning and concept as well as the difficulty level of the items. The two versions were reviewed by two other people who did not know each other who were not involved in the initial translation process and who also were fluent in English. After comparing the translated versions with each other and making minor changes, the final version was prepared. This checklist was translated from Persian to English by two people (one native) to translate. The two translated versions from the target language to the original language were reviewed by the researcher and a translator fluent in Persian and English, and in the final stage, the final version was sent to Mrs. Kolcaba for final approval. Then, the psychometric process of the translated checklist was performed using the determination of face and content validity, construct validity (the known-group technique and PCA), internal consistency (Cronbach’s alpha) and stability reliability (intra-class correlation method).

### Validity and reliability

In the current study, content validity was examined qualitatively and quantitatively. This refers to the extent to which a measure represents all the aspects of the construct being measured. It involves assessing whether the items or questions in a measure represent the measured construct. To check the qualitative content validity [23, 26] the final version of the translated checklist was given to 10 people (two clinical psychologists, two pediatricians, four nursing instructors, and two nursing associates with experience in developing instruments) to choose the most important items of the Comfort Behaviors Checklist and place the items in the correct category, review and provide their corrective opinions. These people were purposefully selected. To examine the validity of quantitative content, the content validity ratio (CVR) and content validity index (CVI) were calculated and reported in the presence of the mentioned experts [27].

Afterward, to examine the qualitative face validity, the translated checklist was given to 10 pediatric nurses selected via a convenient sampling method who had more than five years of experience in nursing children. This refers to the extent to which the measure appears to be measuring what it claims to be measuring. It involves assessing whether the items or questions in a measure seem to be related to the construct being measured. So, their opinions on the appearance characteristics of the checklist were requested.

Regarding the quantitative face validity, the instrument was given to the nurses with an answer sheet containing a 5-point Likert scale (1 = unrelated, 2 = slightly relevant, 3 = relatively relevant, 4 = relevant, and 5 = very relevant) and item impact was calculated [28] and reported by percentage. The item impact score was calculated using the “Item Impact Score = Frequency (%) × Importance” formula, in which frequency means the percentage of people who gave a score of 4 and 5 in face validity and importance is an average of total answers. The acceptability score of each item was considered ≤ 1.5 to evaluate quantitative face validity. If the results were above 1.5, the item remained on the checklist, and if the results were below 1.5, the item was modified to increase the item's importance to above 1.5. If the item was still less than 1.5, the item was removed from the checklist [29].

The known-group technique was used to examine the construct validity [29–32]. This test can discriminate between a group of individuals known to have a particular characteristic and a group who do not have the characteristic [33]. By an 80% Power (1-β error probability), α error probability of 5%, and 0.3 effect size based on G*Power software, 53 sick children hospitalized in the selected hospitals of Tehran, who were less comfortable, were examined and compared to a group of healthy children (53 children), who had sources of comfort and were playing in a kindergarten or a park [34]. Then the Spearman rank correlation coefficient [31] was calculated for intergroup and intragroup of hospitalized and non-hospitalized children, and a hypothesis test [35] was performed, whose results were validated by power analyzing the sample size, as follows:

**Table:** 

**t tests—**Means: Wilcoxon-Mann–Whitney test (two groups)	
**Options:**	A.R.E. method
**Analysis:**	A priori: Compute required sample size
**Input:**	Tail(s) = One
	Parent distribution = Normal
	Effect size d = 0.5
	α err prob = 0.05
	Power (1-β err prob) = 0.80
	Allocation ratio N2/N1 = 1
**Output:**	Noncentrality parameter δ = 2.5152354
	Critical t = 1.6603560
	Df = 99.2225438
	Sample size group 1 = 53
	Sample size group 2 = 53
	Total sample size = 106
	Actual power = 0.8032180

Inclusion criteria for sick children were in the range of 4 to 6 years old, parents' consent to participate in the study, at least six months passed by the diagnosis of chronic disease, no acute illness at the time of the research process, and no recurrence of the disease at the time of visit by the researcher. Healthy 4 to 6-year-old children are considered not to have any disease.

The Principal Component Analysis (PCA) with Oblimin rotation was used to assess construct validity with 220 samples using SPSS version 24. According to the COnsensus-based Standards for the selection of health status Measurement Instruments (COSMIN) checklist, a sample size of seven times the number of items and more than 100 is a very good sample size for factor analysis [36, 37].

Before performing PCA, the univariate normality was checked by skewness (between -3 and + 3 and Kurtosis (between -7 and + 7), and the presence of outliers in the data (by scatterplots and boxplots) was investigated. To decide the number of factors, Scree plots and eigenvalues were used. Bartlett's test result should be significant, and Kaiser–Meyer–Olkin (KMO) > 0.8 is acceptable [38].

To study the reliability of the stability of the Comfort Behaviors Checklist, the inter-rater reliability method was used by calculating the Average-measurement, absolute-agreement, 2-way mixed-effects model [39]. Intraclass Correlation Coefficient (ICC) [31] with the presence of 10 hospitalized children who met the inclusion criteria. The current checklist was filled by two observers who were both nurses working in the pediatric wards with 5 years of experience. A correlation coefficient above 0.70 is acceptable, and a correlation coefficient above 0.75 is considered to be excellent [40, 41]. Afterward, Cronbach's alpha was also calculated to measure the instrument's internal consistency. For the instrument to have sufficient and good internal consistency, Cronbach's alpha should be greater than 0.7.

### Data collection

The researcher referred to the field of research, and after introducing and obtaining permission from the competent authorities of the hospital, she started sampling. In this study, patients with inclusion criteria were selected using a convenient sampling method. Healthy children were also selected according to inclusion criteria. Sampling was performed in the morning and evening shifts without changing the sick child's condition. The Comfort Behaviors Checklist was filled out for healthy children by observing the child while playing in the park.

Qualitative data are described using percentages and quantitative data are reported by mean and standard. Statistical tests such as correlation coefficient were used to determine the construct validity and stability reliability. A significance level less than 0.05 was considered for evaluation. To analyze the present study's data, version 23 of Statistical Package for the Social Sciences (SPSS) software was used.

### Data analysis

Collected qualitative data are described using percentage and frequency, and if quantitative, mean, and standard deviation are used. Statistical tests such as correlation coefficient were used to determine the construct validity and stability reliability. Cronbach’s alpha was also calculated to ensure internal consistency. The significance level is considered to be less than 0.05. To analyze the present study's data, version 23 of SPSS software was used.

## Result

In the current study, the Comfort Behaviors Checklist was filled out for 220 hospitalized children with chronic diseases. The frequency of demographic variables in the research samples is reported in Table 1.

**Table 1:** Frequency of quantitative and qualitative demographic variables of research samples

**Qualitative variables**		**Frequency**	**Percentage (%)**
**Sex**	Female	116	52.7
	Male	104	47.2
**Category of disorder**	Respiratory	21	9.6
	Nervous system and endocrine	29	13.1
	Gastrointestinal	49	22.2
	Cancer	42	19.1
	Metabolic and rheumatoid	62	28.2
	Urology	17	7.8
**Parent education**	High school or lower	86	39.1
	Bachelor or higher	134	60.9
**Quantitative Variables**		**Mean**	**SD ( ±)**
**Child’s age (year)**		5.6	0.46
**Length of stay (day)**		3.8	1.62

### Qualitative and quantitative content validity

After examining the qualitative content validity, some items in the Comfort Behaviors Checklist were changed, which are reported in Table 2. To determine the validity of quantitative content, CVR and CVI were calculated for each item separately. Comparing the CVR results with the Lawshe table, the cut-off point was considered 0.62; Therefore, all 30 items in the draft version of the checklist have an acceptable score. Also, for CVI results, all scores remained on the checklist since the scores were higher than the acceptable cut-off point of 0.8.

**Table 2:** Qualitative content validity

**Domain**	**Item before content validity**	**Item after content validity**
**Vocalization**	Is making moaning sounds	Is Moaning
	Talks meaningfully	Uses meaningful words/sounds
**Motor signs**	Moves fast	Rapid pacing
**Performance**	Enjoys when holding hands	Enjoys touch/hand holding
	Meaningless motions	Has purposeless movements
**Facial Expression**	Looks annoyed	Grimaces/Kicks away
	Too alert	Is too alert
**Other Signs**	Well focused	Is mentally focused
	Able to communicate	Able to have a conversation

### Qualitative and quantitative face validity

According to the nurses' opinions, to examine the qualitative face validity, there was no need to make physical corrections in the structure of the checklist; therefore, the Comfort Behaviors Checklist was confirmed in terms of qualitative face validity. As a result of quantitative face validity as well as the average impact score of each item in the domain, all items scored more than 1.5 and remained on the Comfort Behaviors Checklist.

### Construct validity

53 hospitalized children with chronic disease and 53 healthy children were involved in examining the construct validity using the Known Groups technique. The population of both groups included 27 girls (54%) and 23 boys (46%), and the average age of all these children was 4.5 years old (± 0.76). First, behavioral comfort scores were controlled by the Kolmogorov–Smirnov test (K-S), to check whether the sampling distribution was normal. Due to the non-normality of the data, the nonparametric Spearman rank correlation coefficient was used [42]. Confirmation of the correlation is determined with a significant rate of 0.05. The results are reported in Table 3.

**Table 3:** Correlation of intragroup and intergroup behavioral comfort scores of hospitalized and non-hospitalized children

**Domain**	**Group**	**Spearman’s rho** **(Intragroup)**	***p*-value** **(Intragroup)**	**Spearman’s rho** **(Intergroup)**	***p*-value** **(Intergroup)**
**Vocalizations**	Hospitalized	0.898	0.000	0.253	0.000
	Outpatient	0.829	0.000		
**Motor signs**	Hospitalized	0.838	0.000	0.462	0.002
	Outpatient	0.869	0.000		
**Performance**	Hospitalized	0.950	0.000	0.503	0.000
	Outpatient	0.811	0.000		
**Facial expression**	Hospitalized	0.807	0.000	-0.206	0.007
	Outpatient	0.892	0.000		
**Other Signs**	Hospitalized	0.881	0.000	0.352	0.014
	Outpatient	0.878	0.000		

As shown in Table 3, there is a significant correlation between the intragroup behavioral comfort scores of hospitalized and non-hospitalized children (rho > 0.50, *p*-value ≤ 0.05), indicating the similarity of behavioral comfort scores within each group of children. However, there is a weak correlation between the intergroup behavioral comfort scores of hospitalized children and non-hospitalized children (rho < 0.50, *p*-value ≤ 0.05), suggesting a potential difference between the two groups of children in terms of comfort behavioral scores (Table 3). Then the hypothesis test was performed by the Mann–Whitney U test to examine and confirm the differences in behavioral comfort scores between the two groups (Table 4).

**Table 4:** Testing the hypothesis of the different significance of behavioural comfort list scores in hospitalized and outpatient children with chronic disorders

**Group**	**Frequency**	**Mean**	**SD ( ±)**	**Mean differences**	**df**	**t**	***p*-value**
Hospitalized	53	54.46	12.04	-33.9	48	-13.005	0.000
Outpatient	53	88.36	4.97				

H_0_: Behavioural comfort score in the group of hospitalized children with chronic disorders is greater than or equal to the behavioural comfort score of outpatient children

H_1_: Behavioural comfort score in the group of hospitalized children with chronic disorders is lower than the behavioural comfort score of outpatient children (accepted)

Significance level ≤ 0.05

As shown in Table 4, by confirming the hypothesis of the difference in behavioral comfort scores between the two groups (Table 4), the construct validity is acceptable by the known-groups method.

### Construct validity

The final questionnaire after PCA consisted of 28 items and five factors (two items were removed because of factor loading below 0.3 or cross-loading). Five factors have the eigenvalue above 1. The KMO was 0.877, and Bartlett’s test was 6264.086 (*P* < 0.001) (Table 5). The total explained variance was 70.39%.

**Table 5:** Results of principal factor analysis (*N* = 220)

**Subscales**	**Items**	**Factor loading**	**Communalities**	**Variance explained**
**1**	Has purposeless movements	0.959	0.865	39.382
	Is crying/shouting	0.891	0.951	
	Grimaces/Kicks away	0.872	0.851	
	Rapid pacing	0.823	0.872	
	Is agitated	0.817	0.893	
	Has unusual breathing	0.814	0.806	
	Appears worried/scared	0.796	0.782	
	Is moaning	0.716	0.864	
	Acts anxiously	0.629	0.829	
	Is fidget	-0.617	0.540	
**2**	Accepts kindness	-0.868	0.919	11.712
	Enjoys touch/hand holding	-0.847	0.867	
	Is awake	-0.678	0.495	
	Can eat	-0.550	0.352	
	Has guarding	-0.470	0.711	
**3**	Is peaceful	0.974	0.920	9.412
	Is calm/at ease	0.949	0.865	
	Looks relaxed	0.878	0.740	
	Has relaxed muscles	0.627	0.491	
	Can rest	0.579	0.344	
**4**	Tries to move away	0.834	0.733	5.396
	Looks depressed	0.740	0.560	
	Is rubbing an area	0.712	0.811	
	Is too alert	-0.658	0.736	
	Is complaining	0.504	0.538	
**5**	Smiles	0.693	0.480	4.493
	Able to have a conversation	-0.659	0.488	
	Is mentally focused	0.494	0.407	

### Internal consistency

To assess the internal consistency of the Comfort Behavioural Checklist, the study evaluated its 28 items using the previous sample of 220 participants. The overall Cronbach's alpha coefficient score of the questionnaire was 0.86.

### Stability reliability

The Intraclass Correlation Coefficient (ICC) was calculated by using the inter-rater average measurement, absolute-agreement, 2-way mixed-effects model for scores of 10 children and by two observers. According to Table 6, since the ICC is more than 0.75, the Comfort Behaviors Checklist has an excellent ICC (Table 6).

**Table 6:** Correlation between the behavioural comfort score of a group of hospitalized children

**Mean**	Intraclass Correlation coefficient	Upper Bound	*p*-value
93	0.835	0.961	0.000

After validating the Comfort Behaviors Checklist and ensuring the psychometric dimensions of the instrument, the final version of the Comfort Behaviors Checklist was prepared and filled out for 220 hospitalized children with chronic disorders. At this stage, without nursing intervention, the lowest behavioral comfort score was 47 ± 4.32, and the highest behavioral comfort score was 100 ± 12.34.

## Discussion

The present study suggests that the Comfort Behaviors Checklist may have potential validity and reliability for evaluating the comfort behaviors of children aged 4 to 6 years hospitalized with chronic diseases. The findings indicate that the checklist demonstrates acceptable face and content validity, suggesting that it measures the intended concept appropriately. The acceptable construct validity further suggests that the Comfort Behaviors Checklist accurately measures the underlying construct or concept it is intended to assess by providing meaningful information about it. The acceptable inter-rater reliability indicates a potential level of consistency in the ratings of the checklist. Therefore, healthcare providers could consider using the Comfort Behaviors Checklist as a tool to assess the comfort behaviors of hospitalized children aged 4 to 6 years with chronic diseases.

The General Comfort Questionnaire is a tool designed by Kolcaba to measure comfort in adults [18]. The results of a systematic review by Bosch-Alcaraz et al. also indicate the existence of two instruments for measuring comfort in children in the intensive care unit: the comfort scale and the comfort behavioral scale. The Comfort Behavioral Scale is derived from the Comfort Scale [43]. Both instruments examine distress in children admitted to the intensive care unit. A systematic review by Dorfman et al. in the field of pain, non-pain, related distress, and sedation in children has identified and introduced these two tools [44]. However, based on the researchers' research and correspondence with the designer of the instrument, it seems that no similar study has been conducted on Comfort Behaviors Checklist psychometrics, so it was not possible to compare the results of the present study with similar studies that have examined the psychometrics of this tool.

The General Comfort Questionnaire has two primary forms: long-form (containing 48 items) and short-form (containing 12 items), which assesses general comfort in the form of self-report in physical, spiritual, environmental, and social dimensions [9]. The Comfort Scale also includes eight items that examine the areas of alertness, calmness, respiratory response, movement, mean arterial blood pressure, heart rate, muscle tone, and facial posture [19]. In the present study, 30 behavioral states were examined in Kolcaba's Comfort Behaviors Checklist, which is in the form of 5 dimensions, and none of them were removed after psychometrics.

In assessing the content of the Comfort Behaviors Checklist, all items had a favorable CVR. Also, the present study results showed that the Comfort Behaviors Checklist has acceptable construct validity. In a 2018 study of hemodialysis patients in Indonesia, Artanti et al. examined the validity of the quantitative content of the General Comfort Questionnaire by calculating the CVI and assessed it as acceptable [45]. In 2019, a psychometric assessment of the questionnaire was conducted; 12 new items were added to the tool, which was placed on the two dimensions of fear and anxiety. The validity of the new tool was determined by the content validity method and by calculating CVI, which were acceptable [46]. The item-by-item review of the comfort scale and the construct validity assessment of the scale also questioned three variables in the instrument. Muscle tone, heart rate, and mean arterial blood pressure were relatively less correlated with other variables, and mean arterial blood pressure and heart rate were more correlated than other variables [44]. A study by Grap et al. also showed that comfort scale scores were not significantly associated with the child's predominant behavioral status or behaviors such as leg and head movement and coughing, and their recommendation was to use a combination of individual observations and results from the comfort scale [44, 47] which somehow can cause doubts in the validity of the instrument. However, more psychometric studies are needed to decide on the superiority of the psychometric parameters of Kolcaba's Comfort Behaviors Checklist over the conventional instruments mentioned above, and it is currently not possible to critique due to the lack of psychometric studies.

In 2005, Ferrandiz and Martin conducted a study to translate into Spanish and psychometric evaluation of the General Comfort Questionnaire, calculated a Cronbach's alpha of 0.9, and showed that the questionnaire has an acceptable internal consistency [48]. Also, in 2017, the instrument was examined to measure cultural compatibility and assess its reliability in Brazil. The results showed that this instrument has good internal consistency (Cronbach's alpha 0.8) [49]. The results of the observer agreement also indicate a high interrater agreement in the total score for the comfort scale [44]. The results of other studies have reported high agreement between evaluators on the comfort scale [20, 49]. The interrater agreement for all dimensions of the comfort scale in the study of Van Dijk et al. was higher than 0.6, except for the respiratory response subscale, which was 0.54 [20]. Also, the results of Valkenburg's study showed that the internal consistency of the Comfort Behavioral Scale subscales was more acceptable than the Comfort Scale [50]. In the study of Johansson and Kokinsky, the total score of the interrater agreement of the Comfort Behavioral Scale was higher than 0.7 [ 51]. In the present study, the Comfort Behaviors Checklist had acceptable reliability.

In terms of construct validity, this study could replicate the original five-factor structure of the questionnaire. By removing two items, a five-factor structure was developed. Removed factors were related to verbal communication (item number 4) and other symptoms (item number 30) in the original questionnaire.

The current study has attempted to address the existing gap in measuring comfort in Persian-speaking children by developing and validating a Comfort Behaviors Checklist. Additionally, the study has contributed to the field by examining the psychometric properties of the instrument, which had been lacking in previous studies. These findings highlight the importance of considering the Comfort Behaviors Checklist in future studies as a valid and reliable measure of comfort in children.

This study has its limitations. Failure to evaluate other psychometric parameters, such as feasibility and responsiveness, are the limitations of this study. Another limitation of the study is that different types of validity (e.g., convergent and discriminant validity) could not be investigated, since there is no gold standard for assessing the comfort of children in the Persian version. One other limitation of the current study could be the limited sample size. It is suggested that in future studies, this tool be examined in different and larger populations and checked for its responsiveness and ease of use.

## Conclusion

This study suggests that Kolcaba's Comfort Behaviors Checklist may be a potentially valid and reliable tool for measuring the level of comfort in Iranian children. Using this tool in clinical settings can help measure comfort and ease in children and make decisions to improve comfort and reduce distress in children with chronic diseases. This tool can also provide an accurate and valid assessment of the effectiveness of the intervention in various studies that examine the effectiveness of various interventions in reducing pediatric distress.

## Data Availability

The datasets used and analyzed during the current study are not publicly available in this study but are available from the corresponding author on reasonable request.

## Abbreviations

ICC: Intra-Class Correlation
CVR: Content Validity Ratio
CVI: Content Validity Index
SPSS: Statistical Package for the Social Sciences
